# Slowly cutting, loose seton ligature and staged fistulotomy for healing of idiopathic perianal fistula and influence on anal continence

**DOI:** 10.1007/s00423-023-03005-0

**Published:** 2023-09-07

**Authors:** Lisa Schrader, Birgitte Brandstrup, Gunnar Olaison

**Affiliations:** 1 Department of Surgery, Holbaek Hospital, Part of Copenhagen University Hospitals, Smedelundsgade 60, 4300 Holbaek, Denmark; 2 Department of Clinical Medicine, University of Copenhagen, Blegdamsvej 3B, 2200 Copenhagen N, Denmark

**Keywords:** Perianal fistula, Loose seton, Modified loose seton, Anal continence, Health-related quality of life

## Abstract

**Purpose:**

To investigate the ability of a “slowly cutting, loose seton ligature and staged fistulotomy” to heal perianal fistulas, the time needed with the seton ligature, recurrence rate, influence on anal continence, health-related quality of life (HRQoL), and patient satisfaction.

**Methods:**

Observational single-center study. We reviewed the medical records of all patients with primary surgeries from January 1, 2009, to December 31, 2018. The patients answered a questionnaire pre- and postoperative on anal continence (St. Mark’s incontinence score) and HRQoL (The Short Health Scale). Satisfaction with the operation was answered postoperatively.

**Results:**

Forty-three patients (37 men, 6 women) were included. Initially 41 of 43 healed (95%). Three patients (7%) had a recurrence, two healed after retreatment. The median follow-up was 55 months (IQR, 4). Thirty-four patients (79%) responded to the questionnaire. At follow-up, forty (93%) patients were healed. The median time treated with a seton ligature in the healed patients was 13 months (IQR, 14). St. Mark’s incontinence score preoperative was median 2 (IQR, 9) and after the operation median 1 (IQR, 4). The Short Health Scale improved from median 20 (IQR, 5) preoperatively to 5 (IQR, 5) postoperatively, *p* < 0.001. Patient satisfaction was median 1 (= very satisfied) (IQR, 1).

**Conclusion:**

A “slowly cutting, loose seton ligature followed by a staged fistulotomy”, heals the vast majority of perianal fistulas with minor or none influence on continence and few recurrences. Patient-reported HRQoL improves greatly, and patient satisfaction is high.

## Introduction

Several techniques have beene proposed for the treatment of high anal fistulas, but none have gained the status of gold standard [1]. In the literature, various sphincter-saving techniques have been tested such as advancement flap, the use of biomaterials, plugging, and ligation of the fistula tract in order to avoid sphincter dysfunction. Though the results vary, none of these techniques have promoted healing for a satisfactory number of patients [2–4]. New techniques have come forward including biomaterials, energy devices, and hybrid techniques, but the studies are few, and the results inconclusive. A systematic review concluded that no long-term follow-up data exist for these new techniques, and the evidence to recommend one particular treatment over the others is lacking [5].

The seton ligature has been widely used in the treatment of perianal fistulas and can be traced back to the time of Hippocrates [6]. Presently the seton ligature is used in many ways with both palliative and curative intent. For palliation it promotes drainage before more advanced operations such as advancement flap and plug [3, 4], and it is recommended as the only treatment for fistulas in Crohn’s disease [7]. For curative intent some surgeons remove the ligature after the patient has become asymptomatic [8–10], however, then recurrence become frequent [9, 10]. Moreover, the seton can be tightened (cutting seton), loosely tightened (snug seton) or kept loose, each method differing in pain and outcome for the patient [11–19].

There are observations that even without tightening, in time the seton ligature will migrate towards the skin moving the fistula tract distally to become more superficial with less or no involvement of the sphincter [17–19]. In a minority of cases the seton ligature migrates spontaneously through the skin. For the majority a second operation, a “staged fistulotomy” (lay-open), is needed when there is no longer any involvement of the sphincter. This modification, from here forward referred to as “slowly cutting, loose seton ligature and staged fistulotomy”, appears to preserve fecal continence [17, 18, 20]. It is assumed that when the ligature cuts the sphincter slowly over weeks and months, fibrosis and scarring develop which prevents the anal sphincter fibers from separating and thus preserves sphincter function [9, 17, 18, 20, 21].

We found three studies which used a loosely tied seton ligature  that was allowed to migrate slowly through the sphincter [17, 18, 20]. Treatment time with the seton ligature varied from six weeks up to a year or more. In the patients without spontaneous migration, all the studies used a staged fistulotomy of remaining tissue. The two studies with the longest treatment time reported an initial healing rate of 100% with only minor influence of anal continence [17, 18].

We hypothesized that treatment time with the seton ligature was important, and in order to get the migration as complete as possible, we allowed long treatment time and performed a staged fistulotomy when only the skin or minor non-significant involvement of the sphincters persisted. In this observational study, we present our experience of 43 consecutively treated patients from one surgical department.

## Material and methods

### Design and settings

This study is a single-center, observational study at the Department of Surgery, Holbaek Hospital, Denmark. We collected the data retrospectively from medical records and prospectively by follow-up through a questionnaire.

We performed the primary surgeries between January 1^st^, 2009, and December 31^st^, 2018. The data collection including the questionnaires was carried out November 1^st^, 2019, to June 1^st^, 2020.

The Regional Committee on Health Research Ethics (J.nr. 18–000080), the Danish Data Protection Agency (REG-023–2019) and the Danish Patient Safety Authority (3–3013-2867/1) approved the study. We adhere to the STROBE guidelines for the reporting of the study [22].

### Participants

We identified the patients having surgery for perianal fistulas between January 1^st^, 2009, and December 31^st^, 2018, using the operation codes KJHD20, KJHD23, KJHD30, KJHD33. We screened the medical records for all identified patients for eligibility, and included adult patients (aged ≥ 18 years) with symptomatic, idiopathic anal fistula, who underwent treatment with “slowly cutting, loose seton ligature”. We excluded patients with inflammatory bowel disease, anal or rectal malignancy, anal suppurative hidradenitis, hemorrhoid disease, or anal fissure.

### Variables

We collected the following background data: Age, gender, concomitant diseases, smoking habits, drinking habits, body weight, height, previous anal diseases with treatment, and number of visits in the outpatient clinic. Data related to the fistula treatment: date of surgery, date of lay-open, classification of the fistula according to Parks’ classification [23], time treated with seton ligature, and number of operative revisions. Data on the surgical outcome: Number of patients with healing of the fistula, adverse events graded by the Clavien-Dindo classification [24], and recurrence.

Patients were encouraged to answer a preoperative questionnaire on St. Mark’s incontinence score [25] and again in the postoperative questionnaire. The Short Health Scale [26] adapted for fistulas was assessed pre- and postoperatively from a questionnaire sent at follow-up, which also included patient’s satisfaction with the treatment. If the patients did not respond to the questionnaire, we called the patient by telephone and (if they permitted) filled out the questionnaire by interview.

### Outcome

The primary outcome was the number of patients who healed.

We defined healing as patients free of symptoms with a healed fistula on examination.

We classified the patients without clinical follow-up as healed if they 1. reported no symptoms in the questionnaire or on the telephone, or 2. if they after the staged fistulotomy had no notes on unhealed fistulas in their records.

Secondary outcome measures were time with a seton ligature to healing, incontinence (classified by St. Mark’s incontinence score), recurrence rate, health-related quality of life ((HRQoL) measured with the Short Health Scale), and patient satisfaction with the treatment.

### Procedure

We performed all the operations in general anaesthesia with the patient in the lithotomy position. We examined the anus and the lower rectum using an anoscope and a speculum. We identified the external openings of the fistula(s) and probed the fistula(s) with a tube. In most cases, we could identify the internal opening after probing, sometimes aided by injection of hydrogen peroxide. In some patients, we had to aid the identification of the internal fistula opening with an initial partial excision of the tract. We dissected the fistulous tract in the fibrous sheath surrounding the tract and excised until 3–4 cm from the anus attempting not to cut off sphincter fibers. The seton ligature(s) was placed and loosely tied.

We aimed at having a 3–4 cm long fistula track placing the ligature within the rim between the buttocks, so the patients did not sit on the ligature.

In patients having extensive bilateral fistula systems, we excised one side at a time. We used two setons in each fistula tract. We used either Ethibond® smooth polyester, Ti-Cron® braided polyester, silk braided, or nylon smooth for the seton. In cases of severe infection or abscesses, we incised  the abscesses and added two Medi-loop® rubber bands which were removed after 6–12 weeks. We infiltrated the wound with local anesthetics, and the patients were all discharged from the hospital 4–6 h postoperatively.

We planned follow-ups for all the patients in the outpatient clinic 6–14 weeks postoperatively. Here, we assessed the migration of the seton ligature, the healing of possible abscesses, need for revision, or planning of fistulotomy. We performed the fistulotomy as soon as the seton no longer, or only minimally, involved the sphincters.

### Statistical methods

We used IBM SPSS (version 26, 2019, IBM Corporation, USA) for descriptive statistics and analysis. We tested for normality with either histograms, Q-Q plots or Kolmogorov–Smirnov test. In case of normality, we calculated mean and standard deviation (SD). We present non-normal data as median and interquartile range (IQR) and a Kaplan Meier plot to illustrate the healing time with the seton ligature.

We used the Sign test for differences between pre – and postoperative HrQoL measures. We analysed the difference in incontinence between men and women using the Mann–Whitney-U Test.

The analysis was a per-protocol analysis. We accepted a two-sided *p*-value of 0.05 as significant. We did not replace missing data.

## Results

**Fig. 1:**
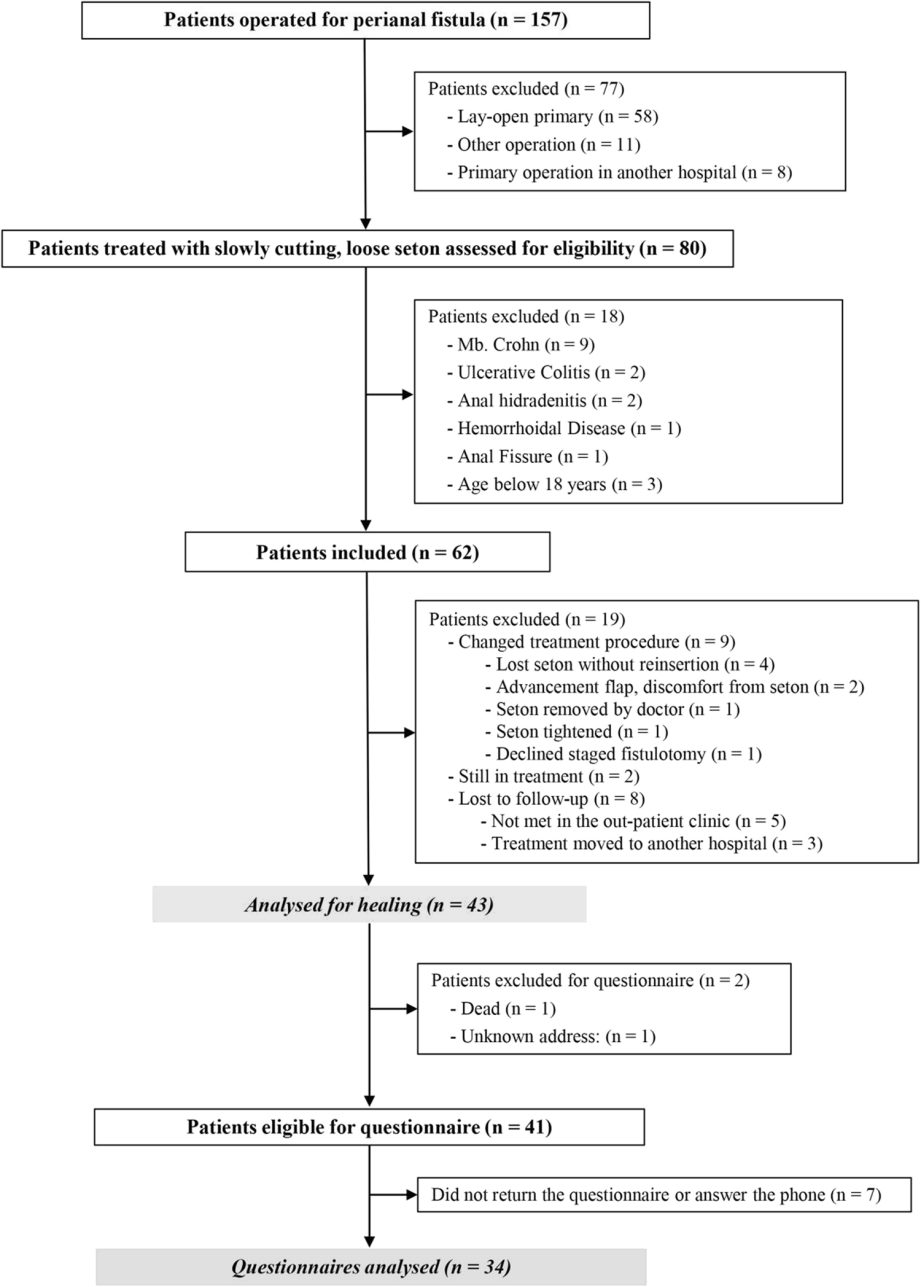
Trial profile

Eighty patients treated with a “slowly cutting, loose seton ligature” were potentially eligible (Fig. 1). Eighteen patients were excluded because of: Crohn’s disease (9), ulcerative colitis (2), hemorrhoid disease (1), active fissure (1), active suppurative hidradenitis (2), or age < 18 years (2). Of the remaining 62 patients, unfortunately 19 patients were: Lost to follow-up (8), in continuous treatment (2), or changed treatment procedure (9) (Fig. 1), leaving 43 patients for the per protocol analysis.

Out of the nine patients who changed treatment procedure, two patients had an advancement flap due to discomfort from the seton and healed, two patients lost their seton and did not want reinsertion despite symptoms, one had the seton removed due to minor fistula symptoms which later recurred, and one had the loose seton changed into a cutting seton at the surgeon’s discretion. The aforementionedhad a fistulotomy and incontinence problems. We have no data on healing for the remaining three patients.

We mailed the questionnaire to the per-protocol population, and 34 patients (79%) responded. The median follow-up time from primary operation until the questionnaire was 55 months (IQR, 4).

Table 1 shows the baseline characteristics for the two populations analysed (the patients analysed for healing and the subgroup that responded to the questionnaire). Included were 37 men and six women, with a mean age of 47 years (SD 14) at operation. The baseline characteristics for the two populations were equally distributed. In the patients analysed for healing 22 (51%) had a trans-sphincteric -, 12 (28%) had an inter-sphincteric -, and nine (21%) had a supra-sphincteric fistula.

**Table 1:** Baseline characteristics for the 43 analysed for healing and the subpopulation of the 34 patients that answered the questionnaire

	Analysed for healing	Analysed questionnaires
No (%)	43 (100)	34 (100)
Age at operation, mean (SD)	46.8 (14.3)	46.2 (2.3)
Women, No (%)	6 (14)	6 (18)
BMI, mean (SD)	27.0 (4.0)	27.0 (0.7)
- Missing, No (%)	3 (7)	2 (6)
Smoking, No (%)	14 (33)	12 (35)
Alcohol overconsumption*, No (%)	4 (9)	4 (12)
Diabetes, No (%)	4 (9)	3 (9)
Current malignancy**, No (%)	3 (7)	1 (3)
Previous fistula surgery, No (%)	3 (7)	2 (6)
- Fistulotomy	2 (5)	2 (6)
- Cutting seton	1 (2)	-
Previous anal fissure, No (%)	2 (5)	1 (3)
Previous perianal abscess, No (%)	31 (72)	24 (71)
Parks’ classification of fistula, No (%)		
- Intersphincteric	12 (28)	10 (29)
- Transsphincteric	22 (51)	18 (53)
- Suprasphincteric	9 (21)	6 (18)

^*^Standard drinks per week > 7 for women and > 14 for men

^**^One prostate cancer, one colorectal cancer and one Non-Hodgkin lymphoma. None of them in chemotherapy

Table 2 shows the operations and adverse events in the 43 patients. Thirty-six (84%) had a staged fistulotomy, six (14%) patients had a spontaneous migration of the seton ligature, and one (2%) patient declined a staged fistulotomy, choosing to keep the seton ligature. The median number of surgical revisions was zero (IQR, 1).

**Table 2:** Operations and adverse events in the 43 patients operated with loose, cutting seton ligature

**Primary operation and staged fistulotomy**	
Loose seton with partial excision of fistula, No (%)	37 (86)
Loose seton only, No (%)	6 (14)
Staged fistulotomy, No (%)*	36 (84)
Spontaneous migration of the seton ligature, No (%)	6 (14)
Adverse Events total, No (%)	3 (7)
- Anal ulcer**	1 (2)
- Bleeding**	1 (2)
- Pain**	1 (2)
**Reinsertion after lost seton ligature**	
Reinsertion, No%	3 (7)
**Surgical revisions**	
Once, No (%)	13 (30)
Twice, No (%)	7 (16)
Tree times, No (%)	3 (7)
Four times, No (%)	2 (5)
Five times, No (%)	1 (2)
Number of surgical revisions, median (IQR)	0 (1)
Adverse Events, No (%)	2 (5)
- Pain**	1 (2)
- Infection^§^	1 (2)
**Number of operations**, median (IQR)	2 (1)

^*^ One patient declined a staged fistulotomy

^**^ Clavien-Dindo grade I. Anal ulcer was conservatively treated and healed. Bleeding was conservatively treated with tamponade. Pain was treated with pain killers

^§^ Clavien-Dindo grade II. Treated with antibiotics

A total of 26 surgical revisions were performed, with the most frequent procedures being shortening of the seton ligature due to displacement over time and discomfort to the patient, revision of a shortened tract, and excision of remaining fistulous tracts in three patients with extensive and/or bilateral tracts, as not all of them were excised initially. These three patients underwent four or five revisions. Other revisions included patients who had initial abscesses drained with a Medi-loop® rubber band, as well as minor skin abscesses that could occur at the external end of the seton ligature.

Among all operations, there were five (12%) registered adverse events. Four were Clavien-Dindo grade I, treated conservatively, and one was a grade II wound infection, which received antibiotics. Information on their treatment is found in Table 2. The median number of visits in the outpatient clinic was four (IQR, 3). Ten (23%) patients had an additional phone consultation.

Table 3 shows the healing outcome after treatment with “slowly cutting, loose seton ligature” in the population of 43 patients. Forty-one (95%) patients healed, and two (5%) did not heal. Both patients who did not heal had a large pararectal cavity, one ended up with a chronic drainage, and the other had an advancement flap operation and healed. Three patients (7%) had a recurrence, two healed after an additional treatment with a “slowly cutting, loose seton ligature”, and one, who was found through the questionnaire, had not sought treatment. At the follow-up, 40 of 43 (93%) patients were healed.

**Table 3:** Healing after treatment with loose, cutting seton ligature in the 43 patients

Analysed for healing No (%)	43 (100)
Healed, No (%)	41 (95)
Time with loose, cutting seton ligature among the healed (months), median (IQR)	13 (14)
Recurrence, No (%)	3 (7)
- Healed after re-treatment with loose, cutting seton ligature, No (%)	2 (5)
- Not re-treated, No (%)	1 (2)
**Healed at the time of analysis**, No (%)	40 (93)

**Fig. 2:**
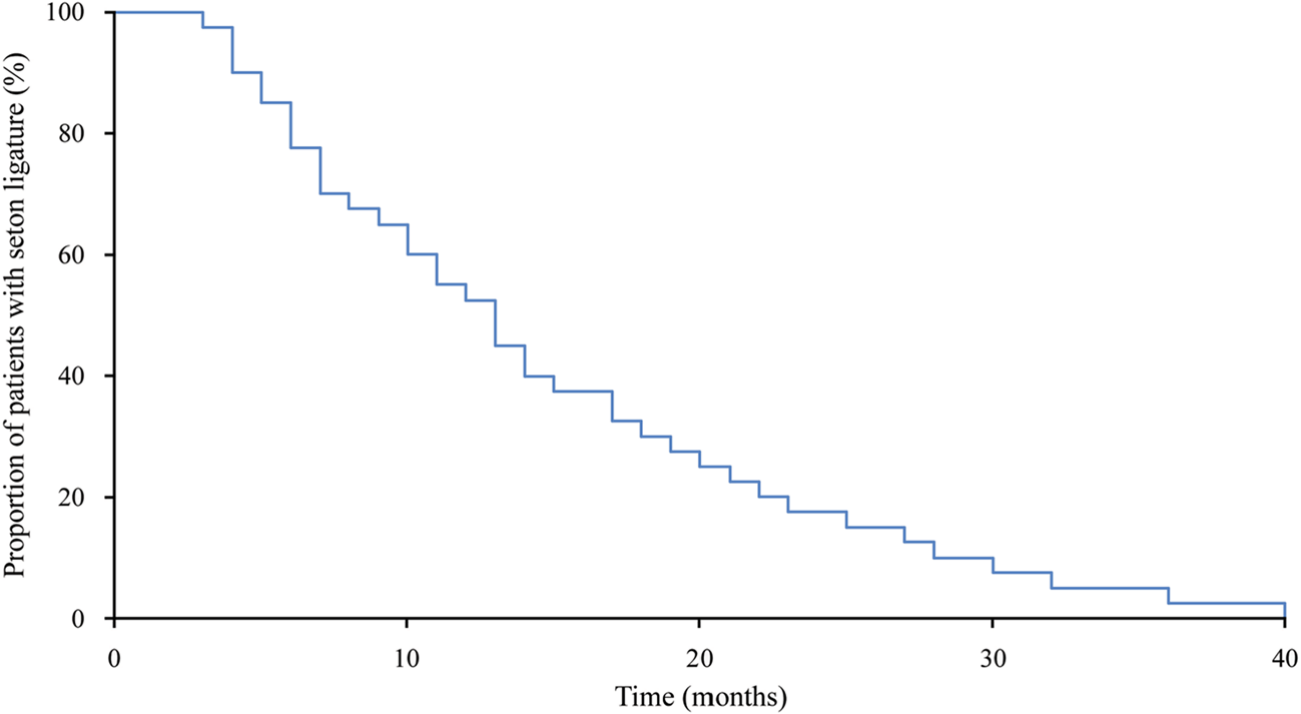
The time treated with a seton ligature in the patients who healed. The median time with the seton ligature was 13 months (IQR, 14). The patient treated for the longest time had a seton ligature for 40 months

Figure 2 shows the time treated with a seton ligature in the patients who healed. The median time with the seton ligature was 13 months (IQR, 14). The patient treated for the longest time had a seton ligature for 40 months.

Preoperative continence in 22 patients according to the St. Mark’s score was 2 (IQR 1) and almost ally due to the use of pads. Table 4 shows postoperative continence and patient satisfaction in the 34 questionnaires analysed. The postoperative median St. Mark’s incontinence score was 1 (IQR, 4) (the score is graded from 0–24, 0 being no symptoms at all). Only one woman, who preoperatively had incontinence problems, reported daily leak of liquid and solid stool. The median patient satisfaction with the treatment was 1 (IQR, 1) (1 being very satisfied and 7 being very dissatisfied).

**Table 4:** Anal continence and patient satisfaction after treatment with loose, cutting seton ligature in the 34 questionnaires analysed

**St. Mark’s incontinence score** (0–24), median (IQR)*	1 (4)
- Missing, No (%)	1 (3)
Incontinence for solid stool, No (%)	4 (12)
- Daily, No (%)	1 (3)
- Sometimes, No (%)	1 (3)
- Rarely, No (%)	2 (6)
Incontinence for liquid stool, No (%)	7 (21)
- Daily, No (%)	1 (3)
- Sometimes, No (%)	2 (6)
- Rarely, No (%)	4 (12)
**Satisfaction with treatment**, median (IQR)**	1 (1)
- Missing, No (%)	2 (6)

^*^ 0 = perfect continence, 24 = totally incontinent. Never = no episodes in the past four weeks; Rarely = 1 episode in the past four weeks; Sometimes =  > 1 episode in the past four weeks but < 1 per week; Weekly = 1 or more episodes a week but < 1 per day; Daily = 1 or more episodes a day

^**^1 = very satisfied, 7 = very dissatisfied

Table 5 shows the Short Health Scale in the 34 questionnaires analysed. The Short Health Scale improved from a median of 21 (IQR, 10) preoperative to 5 (IQR, 5) postoperative (*p* < 0.0001) (4 being best possible).

**Table 5:** Short Health Scale postoperatively and preoperatively answered in retrospect in the 34 questionnaires analysed

Short Heath Scale*	Preoperative	Postoperative	*P*-value
Score (4–28) median (IQR)	21 (10)	5(5)	< 0.0001
- Missing, No (%)**	1 (3)	1 (3)	
- Symptoms (1–7), median (IQR)	6 (3)	1 (1)	< 0.0001
- Function (1–7), median (IQR)	5 (4)	1 (1)	< 0.0001
- Worries (1–7), median (IQR)	5 (3)	2 (4)	< 0.0001
- General well-being (1–7), median (IQR)	4 (3)	1 (1)	< 0.0001

^*^ Short Health Scale consists of 4 symptoms rated on a Likert scale (1 = best, 7 = worst), and the overall score is an addition of the individual scores (4 = best possible, 28 worst possible)

^**^ Preoperative scores were missing for one patient and postoperative scores for another

## Discussion

The present study shows that a “slowly cutting, loose seton ligature followed by a staged fistulotomy” can heal the vast majority of idiopathic perianal fistula, with minor influence on anal continence. Healing rates are in line with previous studies on a cutting seton ligature, but with better outcome for anal continence [11–14]. No other techniques such as advancement flap, plug, LIFT etc. have been able to demonstrate similar healing rates [2, 3].

We found only minor or no influence on anal continence. The long treatment time of about one year might explain this. The treatment time and the staged fistulotomy is most likely important for both recurrence and sphincter function. A per protocol analysis was used which excluded the two patients who had an advancement flap. If they were included as a procedure failure, the healing rate would change from 95 to 91%. However, this change would not have affected the study's overall findings.

In trials tightening the cutting seton ligature at regular intervals, the fistula heals by cutting the sphincters during weeks to months. The healing rates are high [11–14], but so is the risk of fecal incontinence. In a review, the mean incontinence rate was 12% [15].

A “snug seton” (tightened with minimal tension) shows high healing rates in one study, but no formal indices evaluated incontinence. “Minor incontinence” persisted in 25% of patients [16]. Studies using the loose seton without staged fistulotomy find higher recurrence rates: Buchanan et al. had 80% recurrence after long-term follow-up [9], and Eitan et al. had 20% recurrence following a mean of five years follow-up [10]. In both studies, the seton ligature was removed after three to four months.

During the primary operation we found no secondary tracts communicating with the anal canal. However, we suspect that two of the three recurrences may be due to overlooked secondary tracts.

Three other studies have used the “slowly cutting, loose seton ligature” with an approach similar to ours [17, 18, 20]. Kelly et al. is the study most comparable to ours except for their inclusion of IBD patients. Their treatment-time was three months to one year and their healing rate 100%. 6% had recurrence, 4% had minor urgency, and no one had incontinence [17]. Galis-Rozen et al. performed a staged fistulotomy after six to eight weeks, and found a recurrence rate of 47% after two years follow-up. 6% had minor incontinence with soiling [20]. Neither of these two studies used formal indices to measure incontinence. In a recent study by Sungurtekin et al., all patients healed after a treatment-time of two months, there were no recurrences within a follow-up of one to three years, but their patients had a slightly raised Wexner score of 2 and noted at anal manometry a decrease of anal pressure [18].

We used the slowly cutting loose seton and staged fistulotomy to treat some intersphincteric fistulas. The rationale is that these patients all had involvement of the internal sphincter. The slowly cutting seton promotes less injury to the internal sphincter, less scarring of the skin, and keeps the anal ring intact, all factors that may help preserve continence.

We used the St. Mark’s incontinence score [25] to assess continence, which includes urge incontinence. One patient with pre-existing incontinence remained daily incontinent to both solid and liquid stools postoperatively, while a few experienced incontinence rarely or sometimes. Our study reported better continence scores compared to two recent studies using the same score: a randomized study comparing advancement flap with collagen plug [4] and a retrospective study on cutting seton ligature [13].

This is, to our knowledge, the first time the Short Health Scale [26] has been used to evaluate HRQoL after treatment of anal fistulas. The Short Health Scale demonstrated an impressive responsiveness, and was 5 after treatment, which is almost a perfect score. The findings are in accordance with those using generic QoL instruments as Short Form-36 [27] demonstrating improvement after surgery. In addition, the patient satisfaction with the treatment was excellent. The treatment was well tolerated. Only two patients (4%) changed procedure due to discomfort from the seton ligature (Fig. 1).

The strengths of this study are the long-term follow-up, minimizing the risk of overlooking recurrence, and strengthening our finding of minor influence on continence. The validated incontinence score improves the consistency in reporting and the comparability to other studies. A relatively high response rate to the questionnaire gives a reliable prospective follow-up. Our study has limitations: The single-center design limits generalizability. The retrospective design with involvement of several surgeons renders our findings to be only hypothesis generating. The number of surgeons explain the relatively high rate of patients excluded by changed treatment procedure, as some surgeons preferred other treatments, at their own discretion. Moreover, the questionnaire included retrospective questions posing a risk for recall bias.

## Conclusion

We found that a “slowly cutting, loose seton ligature and staged fistulotomy” is a simple, safe, and affordable procedure, associated with excellent healing, minimal influence on continence, excellent patient satisfaction, few recurrences, and few adverse effects. The operation needs further evaluation in controlled, prospective studies using validated measurement instruments. The procedure has a potential to be the treatment option of choice for high anal fistulas.
